# ^18^F-FDG PET/CT metabolic patterns and clinical parameters correlate with disease severity in autoimmune encephalitis

**DOI:** 10.3389/fneur.2026.1880946

**Published:** 2026-09-30

**Authors:** Baocheng Liu, Ning Wang, Weiwei Kong, Yirong Zhu, Jiaxi You, Yanlin Zhang, Yizhen Shi

**Affiliations:** 1 Department of Nuclear Medicine, The Second Affiliated Hospital of Soochow University, Suzhou, China; 2 Department of Nuclear Medicine, Guangyuan First People's Hospital, Guangyuan, China; 3 Department of Neurology, The Second Affiliated Hospital of Soochow University, Suzhou, China

**Keywords:** ^18^F-FDG PET/CT, autoimmune encephalitis, clinical parameters, disease severity, metabolic patterns

## Abstract

**Purpose:**

To investigate the correlation of ^18^F-FDG PET/CT metabolic patterns and clinical parameters with disease severity in patients with autoimmune encephalitis (AE).

**Methods:**

We retrospectively analyzed 35 AE patients who underwent ^18^F-FDG PET/CT at the Second Affiliated Hospital of Soochow University between March 2021 and December 2023. Clinical and auxiliary examination data were collected and analyzed using the *t*-test, Mann–Whitney U test, Fisher’s exact test, and Spearman correlation. Metabolic patterns on PET/CT were assessed both visually and using Cortex ID software across severity groups.

**Results:**

Among the 35 AE patients (21 males, 14 females), 16 were classified as mild and 19 as moderate-to-severe. Compared to the mild group, the moderate-to-severe group exhibited significantly elevated neutrophil percentages and neutrophil-to-lymphocyte ratio (NLR), alongside reduced lymphocyte percentages, lymphocyte counts, and serum calcium ion levels (Z = −2.75 to 2.30; all *p* < 0.05). ^18^F-FDG PET/CT demonstrated a higher abnormality detection rate than brain MRI (*p* < 0.05). The most common imaging finding was hypometabolism in the brain parenchyma, predominantly involving the parietal and occipital lobes. Furthermore, CASE scores demonstrated a negative correlation with Z-scores in the right medial frontal lobe, right superior parietal lobe, and bilateral lateral occipital lobes (*r* = −0.456 to −0.567; all *p* < 0.05).

**Conclusion:**

In addition to clinical symptoms, specific cerebrospinal fluid and serum biomarkers may help evaluate AE severity. ^18^F-FDG PET/CT demonstrates a higher abnormality detection rate than brain MRI, and we preliminarily observed a correlation between regional hypometabolism and clinical severity scores.

## 1 Introduction

Autoimmune encephalitis (AE) encompasses a group of inflammatory disorders of the brain parenchyma mediated by autoimmune mechanisms (1). It presents with acute or subacute onset and diverse clinical manifestations, for which immunotherapy serves as the primary treatment (2). Early and aggressive immunotherapy can mitigate disease severity and is generally associated with favorable outcomes. The severity of acute-phase illness represents a critical prognostic factor in AE (3). According to the diagnostic criteria proposed by Graus et al. (1), the diagnosis of AE relies on a combination of typical clinical symptoms, ancillary test results—including cerebrospinal fluid analysis, electroencephalography, and neuroimaging—as well as autoantibody detection. However, due to the heterogeneity of clinical presentations and inherent limitations of auxiliary examinations, applying these diagnostic criteria in clinical practice remains challenging (4).

Positron emission tomography/computed tomography (PET/CT) has a long-standing history in the evaluation of central nervous system inflammatory diseases. In recent years, ^18^F-FDG PET/CT has emerged as a valuable diagnostic tool in the evaluation of various neurological disorders. By visualizing regional glucose metabolism, it provides crucial insights into brain function and potential abnormalities (5). In early studies, ^18^F-FDG PET was primarily used for the differential diagnosis between infectious encephalitis and non-infectious encephalopathies, confirming its ability to identify the extent of brain involvement in herpes simplex encephalitis at an early stage through alterations in cerebral glucose metabolism patterns (6). With the conceptualization and refinement of autoimmune encephalitis (AE), the application of PET/CT has gradually expanded from “differentiating infection from non-infection” to “localizing and assessing the activity of immune-mediated inflammatory lesions.” Over the past decade, numerous studies have demonstrated that ^18^F-FDG PET/CT can sensitively capture characteristic metabolic abnormalities in the brains of AE patients, with an abnormality detection rate significantly higher than that of magnetic resonance imaging (MRI) (7–9). Patients with AE exhibit heterogeneous cerebral metabolic patterns across different antibody subtypes and disease stages, and no consensus has been reached on a unified cerebral metabolic profile. Furthermore, visual-based subjective interpretation is highly dependent on the experience of readers. Therefore, in-depth analysis of PET/CT images from AE patients is required to improve the auxiliary diagnostic performance of PET/CT. Voxel-based semi-quantitative analyses (e.g., SPM), which can accurately localize brain regions with abnormal metabolism, have been widely adopted (10). This study aims to investigate the characteristics of ^18^F-FDG PET/CT and clinical parameters in AE patients across different levels of disease severity, with the goal of facilitating early diagnosis, supporting timely therapeutic intervention, and aiding in the prediction of disease outcomes.

## 2 Methods and materials

### 2.1 Study population

A retrospective analysis was conducted on patients diagnosed with autoimmune encephalitis (AE) who underwent ^18^F-FDG PET/CT during hospitalization at the Second Affiliated Hospital of Soochow University between March 2021 and December 2023 (Figure 1).

**Figure 1:**
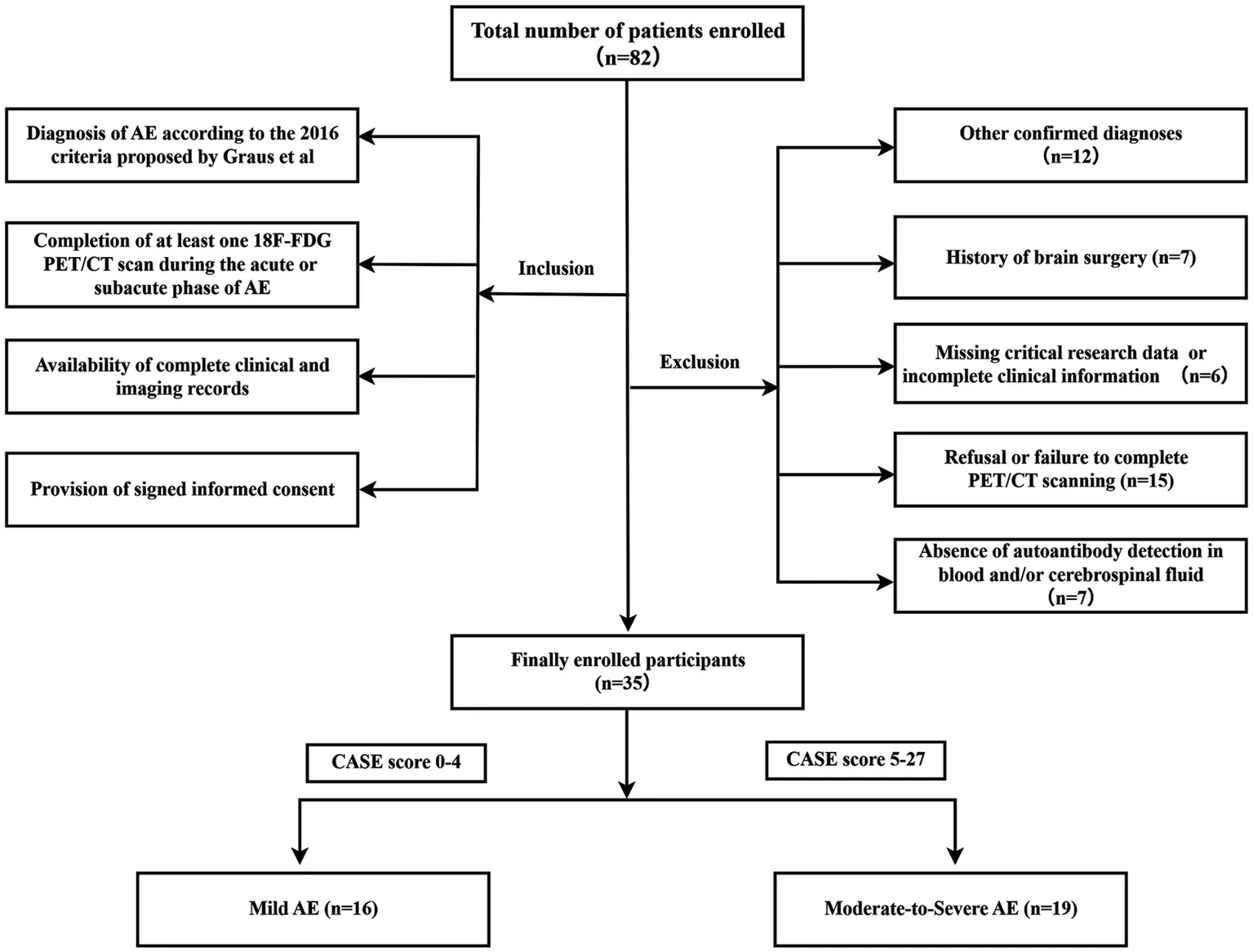
Autoimmune encephalitis patient enrollment flowchart.

#### 2.1.1 Inclusion criteria

1. Diagnosis of AE according to the 2016 criteria proposed by Graus et al. (1).
2. Completion of at least one ^18^F-FDG PET/CT scan during the acute or subacute phase of AE.
3. Availability of complete clinical and imaging records.
4. Provision of signed informed consent.

#### 2.1.2 Exclusion criteria

1. Other confirmed diagnoses such as Alzheimer’s disease, Parkinson’s disease, central nervous system tumors, or cranial trauma.
2. History of brain surgery.
3. Missing critical research data or incomplete clinical information.
4. Refusal or failure to complete PET/CT scanning.
5. Absence of autoantibody detection in blood and/or cerebrospinal fluid.

According to the Graus diagnostic criteria (1), patients were categorized into two groups: ① antibody-positive autoimmune encephalitis (AE): detection of autoimmune neuronal antibodies in the cerebrospinal fluid (CSF) and/or serum, including definite AE, antibody-positive limbic encephalitis, and anti-NMDA receptor (NMDAR) encephalitis; ② antibody-negative AE: diagnosis required the simultaneous fulfillment of the following three criteria: no known neuronal antibodies detected in the CSF/serum; presence of at least one of the following manifestations (specific oligoclonal bands in the CSF, intrathecal synthesis of immunoglobulins or elevated CSF IgG index, pleocytosis or elevated protein levels in the CSF, brain MRI abnormalities suggestive of AE, or responsiveness to immunotherapy); and reasonable exclusion of other etiologies. Antibody-negative AE was further subclassified into antibody-negative limbic encephalitis, probable AE, and possible AE. Two experienced neurologists independently reviewed the medical records in a blinded manner and completed the subclassification of patients.

Disease severity was assessed using the Clinical Assessment Scale for Autoimmune Encephalitis (CASE). Patients were categorized into two groups: a mild group (CASE score 0–4) and a moderate-to-severe group (CASE score 5–27) (4, 11). The CASE score was independently evaluated and classified by two experienced neurologists, both of whom were blinded to the PET/CT and MRI imaging results. The inter-rater reliability was ICC = 0.92 (95%CI: 0.86–0.96, *p* < 0.001). Any disagreements between the two raters were resolved by a third senior neurologist to reach a final consensus.

### 2.2 Clinical data collection

Collected data included patient name, age at onset, gender, clinical manifestations, routine hematological indices at admission, cerebrospinal fluid test results, MRI and ^18^F-FDG PET/CT imaging findings, electroencephalogram (EEG) records, and treatment details.

#### 2.2.1 Autoimmune antibody testing

All patients underwent autoimmune antibody testing in serum and/or cerebrospinal fluid (CSF), including antibodies against N-methyl-D-aspartate receptor (NMDAR), leucine-rich glioma-inactivated 1 (LGI1), contactin-associated protein 2 (CASPR2), γ-aminobutyric acid B receptor (GABABR), α-amino-3-hydroxy-5-methyl-4-isoxazolepropionic acid receptor (AMPAR), dopamine 2 receptor (D2R), metabotropic glutamate receptor 5 (mGluR5), IgLON family member 5 (IgLON5), and glutamate decarboxylase 65 (GAD65), as well as classic paraneoplastic antibodies (Hu, Yo, Ri, MA2, CV2, amphiphysin). Autoimmune antibody testing in serum and/or CSF was performed using cell-based assays (CBA), tissue-based assays (TBA) biochip methodology, and indirect immunofluorescence.

#### 2.2.2 Brain MRI equipment and scanning protocol

Brain MRI was performed using a 3.0 T Philips Achieva MR scanner (Philips, Best, Netherlands) equipped with a 32-channel phased-array head coil. Images were acquired using T1-weighted imaging (T1WI), T2-weighted imaging (T2WI), fluid-attenuated inversion recovery (FLAIR), and diffusion-weighted imaging (DWI) sequences. All subjects were examined in the supine position with the head stabilized, and the midsagittal plane aligned with the coil center. MRI scanning parameters were as follows: sagittal acquisition; TR = 7.1 ms; TE = 3.5 ms; acquisition matrix = 220 × 199; reconstruction matrix = 352 × 352; flip angle = 8°; FOV = 220 mm^2^; slice thickness = 1.0 mm; slice gap = 0 mm; number of slices = 155; scan duration = 3 min. Brain MR images were independently interpreted by two specialized radiologists. MRI abnormalities were defined as the presence of definite AE-related findings on T2WI and FLAIR sequences. Age-related white matter changes, lacunar infarcts, brain atrophy, and other non-specific findings were excluded from the analysis.

#### 2.2.3 ^18^F-FDG PET/CT equipment and scanning protocol

All patients underwent whole-body and brain tomographic imaging on a GE Discovery MI PET/CT system. Patients fasted for more than 6 h prior to the examination, with fasting blood glucose levels ≤ 8.0 mmol/L (for diabetic patients, fasting blood glucose was ≤11.1 mmol/L). Patients did not use sedatives, glucocorticoids, or antiepileptic drugs within 24 h before the scan. Each patient received an intravenous bolus injection of 0.1 mCi/kg (3.7 MBq/kg) of ^18^F-FDG (provided by Shanghai Atom Kexing Pharmaceutical, with radiochemical purity ≥ 90%) over 1 min. Patients were then asked to rest in a dedicated room, avoiding light and noise, for 60 min before image acquisition. The scan ranged from the skull vertex to the mid-femur. A low-dose helical CT scan was performed first (CT parameters: tube voltage 120 kV, tube current automatically modulated between 150 and 250 mA, slice thickness 3.75 mm), followed by a whole-body PET scan (PET parameters: 6–8 bed positions, 2 min per bed position), and then a separate approximately 12 min dedicated brain PET scan. The CT data from the same scanner were used for PET image registration and attenuation correction. CT and PET images were transmitted to a post-processing workstation (GE Healthcare Advantage Workstation, AW version 4.7) to generate axial, coronal, and sagittal CT images and three-dimensional projection images. The equipment underwent daily standardized quality control (including uniformity, resolution, and count rate calibration) and received annual performance validation from nationally accredited metrology institutions.

### 2.3 Image analysis

#### 2.3.1 Visual analysis

Interpretation of ^18^F-FDG PET/CT images was performed independently and in a blinded fashion by two experienced nuclear medicine physicians, who were completely unaware of the patients’ antibody results, CASE scores, and clinical grouping. Inter-rater reliability was κ = 0.89 (95% CI: 0.78–1.00, *p* < 0.001). Any disagreements between the two readers were resolved by a third senior attending nuclear medicine physician. Metabolic abnormalities were defined as regions showing clearly increased or decreased FDG uptake across at least two consecutive cortical slices within a brain lobe. MRI images were assessed by two specialized radiologists. Abnormalities were identified as T2 or FLAIR hyperintensities in the limbic system (unilateral or bilateral) or other brain regions, while excluding age-related white matter changes, lacunar infarcts, and other nonspecific lesions.

#### 2.3.2 Software-based analysis

The Cortex ID application embedded in the GE AW workstation was used to analyze cerebral ^18^F-FDG uptake. The Z-scores used in this study were generated automatically by the Cortex ID software integrated into the GE Healthcare Advantage Workstation (AW version 4.7). This software employs GE’s Cortex ID normative database of brain metabolism in healthy individuals, which was constructed on the basis of a large sample of healthy volunteers. The database includes individuals without central nervous system disorders, psychiatric diseases, structural brain lesions, or systemic conditions that affect cerebral glucose metabolism (e.g., diabetes mellitus, thyroid dysfunction). It has a balanced sex distribution and covers an age range of 18–80 years, stratified by 10-year intervals. Depending on the patient’s age, the software automatically matches the corresponding age-specific metabolic baseline, thereby eliminating the confounding effect of age-related physiological changes in brain metabolism. Z-score calculation is performed against the full normative database using whole-brain grey-matter average metabolism as the internal reference. A brain region was considered significantly abnormal if the absolute Z-score exceeded 2 (hypometabolism: Z < − 2; hypermetabolism: Z > 2).

## 3 Statistical analysis

Statistical analyses were performed using SPSS version 27.0. The Shapiro–Wilk normality test was used to determine whether measurement data followed a normal distribution. Normality was examined using the Shapiro–Wilk test. Variables with *p* > 0.05 were regarded as normally distributed and analyzed with parametric tests. Between-group comparisons were performed using the independent-samples *t*-test, and data were expressed as the mean ± standard deviation (Mean ± SD). Variables with *p* ≤ 0.05 were considered non-normally distributed and analyzed with non-parametric tests. Between-group comparisons were conducted using the Mann–Whitney U test, and data were presented as the median (interquartile range) [M (IQR)]. Fisher’s exact test for categorical variables expressed as frequencies (percentages), and Cohen’s kappa coefficient was used to assess agreement. Correlations between disease severity and other variables were assessed using Spearman’s rank correlation coefficient. A two-sided *p*-value < 0.05 was considered statistically significant.

## 4 Results

A total of 35 patients were finally enrolled, including 21 males and 14 females, with a mean age of 50.66 ± 19.06 years. All patients underwent serum and cerebrospinal fluid (CSF) antibody testing (35/35, 100%). Among them, 19 cases (54.3%) were diagnosed with antibody-negative autoimmune encephalitis (AE), and relevant antibodies were detected in the serum or CSF of 16 cases. Specifically, characteristic antibodies for definite AE were identified in 12 cases (34%), including anti-NMDAR in 4 cases, anti-GFAP in 3 cases, anti-YO in 2 cases, anti-mGluR in 1 case, anti-CV2 in 1 case, and anti-GAD65 in 1 case.

### 4.1 Clinical parameters analysis results

Among the 35 enrolled patients, 16 (45.7%) were classified into the mild AE group and 19 (54.3%) into the moderate-to-severe AE group. Compared with the mild group, the moderate-to-severe group exhibited a higher prevalence of seizures, memory impairment, speech disorders, and gait instability. In terms of laboratory findings, the moderate-to-severe group showed significantly higher neutrophil percentage and neutrophil-to-lymphocyte ratio (NLR), along with lower lymphocyte percentage, lymphocyte count, and serum calcium ion concentration. The differences between the two groups were statistically significant (all *p* < 0.05; Table 1).

**Table 1:** Clinical characteristics of AE patients with different disease severity.

Characteristics	Mild AE (*N* = 16)	Moderate–Severe AE (*N* = 19)	Difference between groups (95% CI)	*p*-value
M/F	10/6	11/8	4.6% (−27.9 to 37.1%)/−4.6% (−37.1 to 27.9%)	1.000
Age (yrs.)	52.4 ± 14.9	49.2 ± 22.3	3.2 (−10.1 to 16.5)	0.632
Length of stay (*n*)	13.5 (8.5, 22.8)	21 (14, 26)	7.5 (0.8 to 14.2)	0.361
Symptoms, *n* (%)				
Seizures	0 (0%)	8 (42.1%)	42.1% (20.2 to 64.0%)	0.004*
Visual hallucinations	1 (6.3%)	6 (31.6%)	25.3% (1.2 to 49.4%)	0.096
Memory impairment	3 (18.8%)	11 (57.9%)	39.1% (9.6 to 68.6%)	0.036*
Neuropsychiatric symptoms	4 (25%)	7 (36.8%)	11.8% (−18.5 to 42.1%)	0.493
Sleep disturbance	3 (18.8%)	7 (36.8%)	18.0% (−10.8 to 46.8%)	0.285
Speech disorder	3 (18.8%)	11 (57.9%)	39.1% (9.6 to 68.6%)	0.036*
Consciousness disorder	6 (37.5%)	13 (68.4%)	30.9% (−0.9 to 62.7%)	0.095
Gait instability	3 (18.8%)	11 (57.9%)	39.1% (9.6 to 68.6%)	0.036*
Blood laboratory parameters				
Neutrophil count, 10^9^/L	4.76 ± 2.23	6.40 ± 2.79	1.64 (−0.12 to 3.40)	0.067
Neutrophil percentage, %	63.3 (53.65, 74.45)	80.8 (73.8, 87.9)	17.5 (8.2 to 26.8)	0.023*
Lymphocyte count, 10^9^/L	1.9 (1.2, 2.5)	1.0 (0.85, 1.3)	−0.9 (−1.4 to −0.4)	0.016*
Lymphocyte percentage, %	30 (18.75, 37.68)	12.6 (8.4, 20.5)	−17.4 (−25.8 to −9.0)	0.011*
NLR	2.1 (1.4, 4.35)	6.5 (4.1, 10.5)	4.4 (1.9 to 6.9)	0.006*
T-lymphocyte percentage, %	68.93 ± 9.40	65.84 ± 11.70	−3.09 (−10.4 to 4.2)	0.402
B-lymphocyte percentage, %	14.47 ± 5.59	16.53 ± 9.96	2.06 (−3.6 to 7.7)	0.467
NK cell percentage, %	14.81 ± 8.09	14.73 ± 7.26	−0.08 (−5.3 to 5.2)	0.976
Sodium concentration, mmol/L	142.1 (140.1, 143.6)	139.4 (136.7, 144.3)	−2.7 (−5.8 to 0.4)	0.159
Potassium concentration, mmol/L	3.97 ± 0.38	3.82 ± 0.43	−0.15 (−0.43 to 0.13)	0.264
Chloride concentration, mmol/L	104 (102.3, 107)	104.3 (103.5, 107.5)	0.3 (−2.0 to 2.6)	0.585
Calcium concentration, mmol/L	2.25 ± 0.15	2.13 ± 0.17	−0.12 (−0.22 to −0.02)	0.028*
Phosphorus concentration, mmol/L	1.16 (1.08, 1.22)	1.09 (1.01, 1.16)	−0.07 (−0.15 to 0.01)	0.353
Magnesium concentration, mmol/L	0.90 (0.86, 0.95)	0.87 (0.79, 0.96)	−0.03 (−0.09 to 0.03)	0.466
Thyroid dysfunction, *n* (%)	4 (25%)	8 (42.1%)	17.1% (−14.1 to 48.3%)	0.476
Positive autoantibody screening, *n* (%)	5 (31.3%)	9 (47.4%)	16.1% (−15.6 to 47.8%)	0.491
Abnormal tumor markers, *n* (%)	5 (31.3%)	8 (42.1%)	10.8% (−20.6 to 42.2%)	0.727
CSF findings				
CSF pressure, mm H_2_O	131.5 (118.8, 192.5)	170 (137.5, 200)	38.5 (−4.8 to 81.8)	0.081
CSF leukocyte count, 10^6^/L	7.5 (2, 37.5)	15 (5, 27)	7.5 (−9.2 to 24.2)	0.550
CSF total protein, mg/L	431.5 (387.3, 813)	483 (274, 964.5)	51.5 (−190 to 293)	0.208
CSF Albumin, mg/L	250 (202.2, 525.4)	316.3 (134.2, 620)	66.3 (−164 to 297)	0.354
CSF IgG, mg/L	42.9 (32.3, 74.2)	51.6 (26.1, 141)	8.7 (−27 to 44.4)	0.573
CSF IgA, mg/L	6.21 (1.67, 10.5)	6.31 (2.17, 13.8)	0.1 (−4.4 to 4.6)	0.643
CSF IgM, mg/L	0.87 (0.35, 2.69)	1.17 (0.64, 1.81)	0.3 (−0.8 to 1.4)	0.446
CSF glucose, mmol/L	3.68 (2.74, 4.06)	3.70 (3.53, 5.51)	0.02 (−0.8 to 0.84)	0.220
CSF chloride, mmol/L	126.3 (125.7, 128.2)	126.5 (124.6, 128.5)	0.2 (−1.8 to 2.2)	0.679
Auxiliary examinations, *n* (%)				
Abnormal EEG	7/12 (58.3%)	15/16 (93.8%)	35.5% (4.2 to 66.8%)	0.057
Abnormal MRI findings	7/15 (46.7%)	3/19 (15.8%)	−30.9% (−59.8% to −2.0%)	0.068
Abnormal ^18^F-FDG PET/CT findings	12 (75%)	18 (94.7%)	19.7% (−3.2 to 42.6%)	0.156
Treatment modality, *n* (%)				
No immunotherapy	2 (12.5%)	0 (0%)	−12.5% (−28.7 to 3.7%)	0.202
Steroid monotherapy	4 (25%)	3 (15.8%)	−9.2% (−35.7 to 17.3%)	0.677
IVIg monotherapy	1 (6.3%)	0 (0%)	−6.3% (−18.1 to 5.5%)	0.457
Corticosteroids combined with IVIg	9 (56.3%)	14 (73.7%)	17.4% (−13.3 to 48.1%)	0.311
Second-line agent monotherapy	0 (0%)	2 (10.5%)	10.5% (−3.2 to 24.2%)	0.489

*p < 0.05 was considered statistically significant.

As shown in Table 1, 12.5% (2/16) of patients in the mild group received no immunotherapy, whereas none of the patients in the moderate-to-severe group remained treatment-naive. Patients with moderate-to-severe AE tended to receive more aggressive regimens, with a higher proportion receiving combined high-dose corticosteroids plus intravenous immunoglobulin (IVIg) (73.7% vs. 56.3%). Second-line immunotherapeutic agents were used exclusively in the moderate-to-severe subgroup (2/19, 10.5%). Although none of these inter-group differences reached statistical significance (all *p* > 0.05).

Regarding the timing of immunotherapy relative to PET/CT, the median interval from PET/CT to immunotherapy initiation across the entire cohort was 1 (IQR − 1 to 5) day; a negative value indicates that the PET/CT scan was performed after immunotherapy had been started. Of the 35 patients, 21 (21/35, 60%) had already initiated immunotherapy prior to PET/CT, whereas 14 (14/35, 40%) had not. For the mild subgroup, the median interval was 3 (IQR − 0.25 to 6) days; for the moderate-to-severe subgroup, the median interval was 1 (IQR − 1 to 4) day, with no statistically significant difference between the two subgroups (*p* = 0.423).

### 4.2 Visual analysis results

Among the 35 patients included in the study, the median time from symptom onset to brain MRI was 22 (IQR 12–40) days for the 34 patients who underwent the examination, and the median time from onset to brain ^18^F-FDG PET/CT was 26 (IQR 16–50) days for the 35 patients. There was no statistically significant difference in the interval between MRI and PET/CT scans for the enrolled patients (*p* = 0.400). Spearman correlation analysis showed no significant correlation between the time interval from symptom onset to PET/CT acquisition and the CASE score (*r* = 0.094, *p* = 0.593). For the 34 patients with both MRI and PET/CT, the median time lag (PET date minus MRI date) between MRI and PET/CT was 4 (IQR 1 to 8) days; 85.3% (29/34) of patients underwent PET/CT after MRI. Subgroup analysis stratified by imaging findings: among 20 patients with PET-abnormal but MRI-normal findings, median MRI-to-PET interval was 3 (IQR: 1 to 7.5) days; for 4 patients with both normal PET and MRI, median interval was 5 (IQR 2.75 to 8.25) days; for 10 patients with abnormalities on both modalities, median interval was 3.5 (IQR − 2.75 to 8.25) days. No significant differences were detected between PET-abnormal/MRI-normal patients and the other two groups (*p* = 0.371 and *p* = 0.684).

Abnormal MRI findings were observed in 10 cases (10/34, 29.4%), with the most common abnormality being T2/FLAIR hyperintensity in the medial temporal lobe. In contrast, 30 patients (30/35, 85.7%) exhibited significant cerebral metabolic abnormalities on ^18^F-FDG PET/CT. ^18^F-FDG PET/CT demonstrates a higher abnormality detection rate than brain MRI (*p* < 0.05). The most frequent hypometabolic regions included the parietal lobe (22/30, 73.3%), occipital lobe (20/30, 66.7%), frontal lobe (20/30, 66.7%), and temporal lobe (18/30, 60%). Hypermetabolism was most commonly observed in the basal ganglia (11/30, 36.7%). The overall positivity rate of ^18^F-FDG PET/CT was 75% (12/16) in the mild AE group and 94.7% (18/19) in the moderate-to-severe group, though this difference was not statistically significant (*p* = 0.156). However, the moderate-to-severe group demonstrated a significantly higher prevalence of multifocal brain involvement. Comparative analysis revealed that hypometabolism in the frontal, parietal, and occipital lobes was significantly more common in the moderate-to-severe group than in the mild group (all *p* < 0.05). The anatomic distribution and frequency of lesions are summarized in Figure 2 and representative imaging cases are provided in Figure 3.

**Figure 2:**
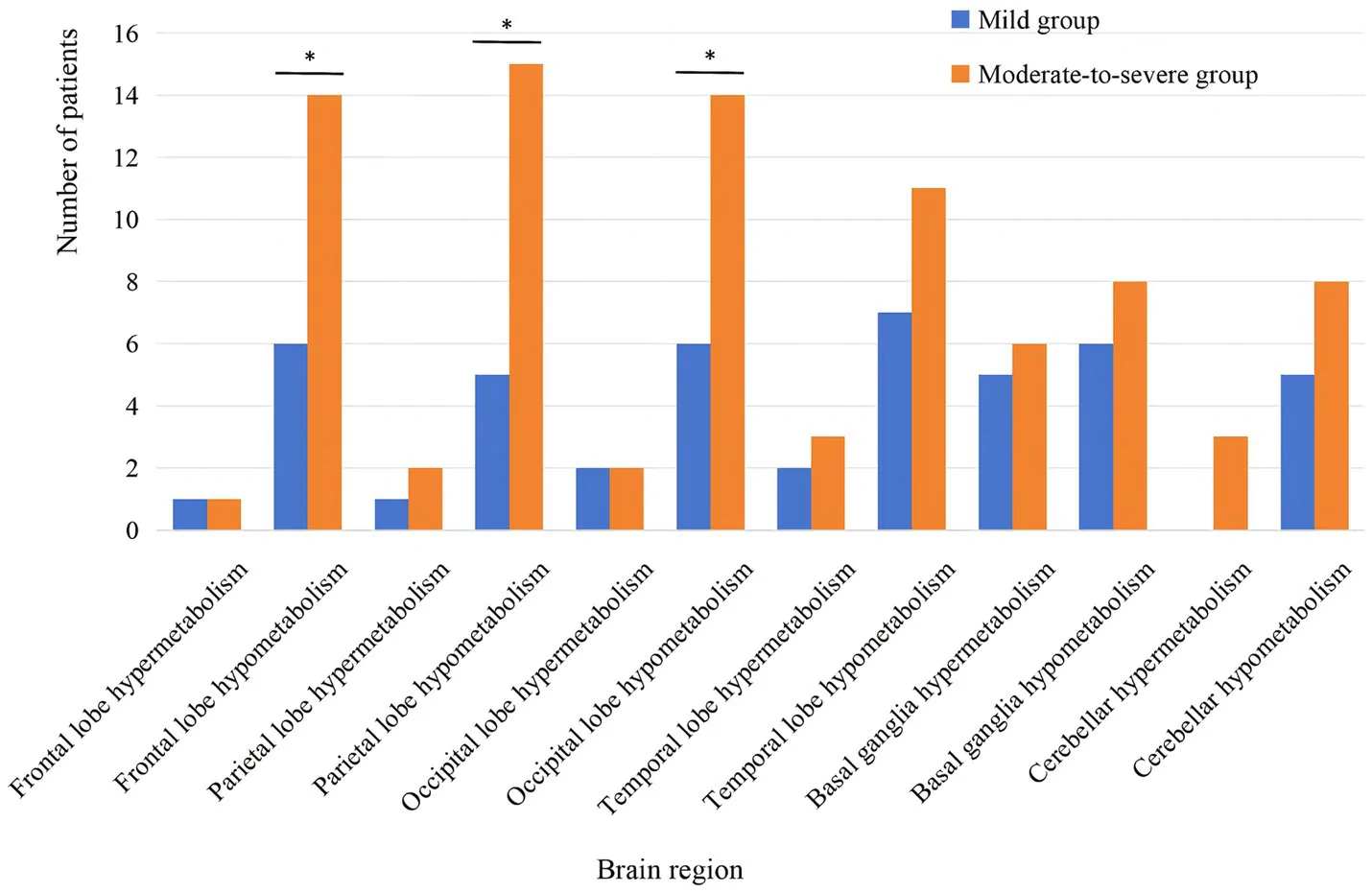
Visual analysis of ^18^F-FDG PET/CT in AE patients with different disease severities (* *p* < 0.05).

**Figure 3:**
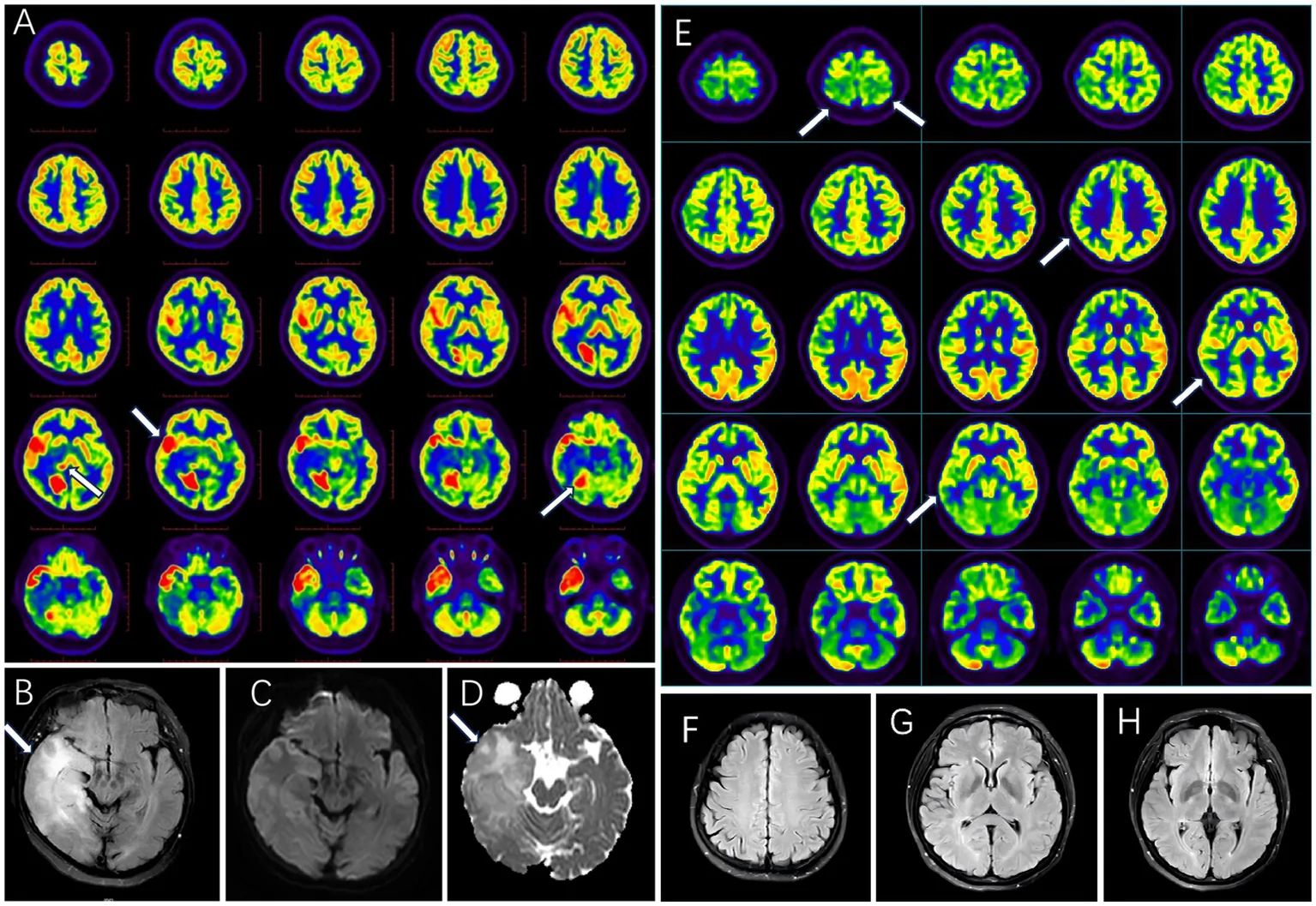
Brain ^18^F-FDG PET/CT and MRI findings in two patients with autoimmune encephalitis. **(A–D)** A 57-year-old woman with seronegative autoimmune encephalitis (CASE score: 6, moderate-to-severe). She was admitted with nausea, vomiting, and epileptic seizures. Brain ^18^F-FDG PET/CT **(A)** showed focal patchy and linear hypermetabolism involving the right temporal lobe, right occipital lobe, and right thalamus, with mild hypometabolism in the remaining cerebral parenchyma. Brain MRI **(B–D)** demonstrated extensive T2-weighted/FLAIR hyperintensities and DWI/ADC signal abnormalities in the right temporal lobe and deep hippocampal regions. **(E–H)** A 31-year-old woman with anti-NMDAR antibody-positive autoimmune encephalitis (CASE score: 7, moderate-to-severe). She presented with a one-week history of speech disturbance, memory impairment, and seizures. Brain ^18^F-FDG PET/CT **(E)** revealed marked hypometabolism in the bilateral frontal lobes, right occipital lobes, right parietal lobe, and right temporal lobe (arrows). Brain MRI **(F–H)** using T2-weighted/FLAIR sequences showed no remarkable parenchymal abnormalities.

### 4.3 Cortex ID software analysis results

Quantitative analysis using Cortex ID software revealed abnormal cerebral metabolic patterns in 31 out of 35 patients (88.6%), a detection rate slightly higher than that of visual analysis (85.7%), though the difference was not statistically significant (*p* = 0.477). The diagnostic consistency between the two analysis methods was κ = 0.72 (95% CI: 0.54–0.90, *p* < 0.001). According to Cortex ID, the most frequently affected regions of hypometabolism were the parietal lobe (23/35, 65.7%), occipital lobe (20/35, 57.1%), and frontal lobe (17/35, 48.6%). Hypermetabolism was most commonly observed in the temporal lobe (8/35, 22.9%).

Cortex ID analysis revealed that both the mild and severe disease groups primarily exhibited reduced cerebral parenchymal metabolism; however, there was no statistically significant difference between the two groups regarding the affected brain regions (all *p* > 0.05). Nevertheless, the Z-scores for the right medial frontal lobe, right superior parietal lobe, and bilateral lateral occipital lobes were lower in the severe disease group compared to the mild disease group, and these differences were statistically significant (all *p* < 0.05).

To evaluate whether PET scan timing influenced metabolic patterns, patients were stratified using the median interval from symptom onset to PET/CT (26 days) as the cut-off, yielding an early-scan group (≤26 days, *n* = 18) and a late-scan group (>26 days, *n* = 17). The overall rate of abnormal PET findings was 94.4% (17/18) in the early-scan group versus 76.5% (13/17) in the late-scan group (*p* = 0.177). No significant inter-group differences were observed in the frequency of lobar hypometabolism for the frontal (55.6% vs. 58.8%), parietal (66.7% vs. 58.8%), occipital (61.1% vs. 52.9%), or temporal lobes (61.1% vs. 47.1%) (all *p* > 0.05). The prevalence of basal-ganglia hypermetabolism was 38.9% (7/18) in the early-scan group and 23.5% (4/17) in the late-scan group (*p* = 0.471). In addition, no significant differences in Cortex ID-derived cortical Z-scores were detected between the two groups for any brain region (all *p* > 0.05).

### 4.4 Correlation analysis between cortex ID Z-score and disease severity

Spearman correlation analysis demonstrated a significant negative correlation between the CASE score and Z-scores in several brain regions, among the included AE patients, the CASE score showed a negative correlation with the Z-score in the right medial frontal gyrus (*r* = −0.456, 95% CI: −0.657 to −0.0745; *p* = 0.006), the right superior parietal lobe (*r* = −0.550, 95% CI: −0.751 to −0.256; *p* < 0.001), the right lateral occipital lobe (*r* = −0.567, 95% CI: −0.761 to −0.278; *p* < 0.001), and the left lateral occipital lobe (*r* = −0.555, 95% CI: −0.754 to −0.262; *p* = 0.001). To further validate the robustness of these findings against potential confounding by pre-scan immunotherapy, we performed a partial correlation analysis adjusting for pre-PET/CT immunotherapy status as a binary covariate (treated vs. untreated prior to scanning). After adjustment, the negative correlations remained statistically significant: right medial frontal lobe (partial r = −0.446; 95% CI: −0.662 to −0.072; *p* = 0.010), right superior parietal lobe (partial r = −0.582; 95% CI: −0.752 to −0.325; *p* < 0.001), right lateral occipital lobe (partial r = −0.463; 95% CI: −0.773 to −0.205; *p* = 0.008), and left lateral occipital lobe (partial r = −0.519; 95% CI: −0.741 to −0.221; *p* = 0.002). These results suggest that the association between regional cortical hypometabolism and disease severity persists after accounting for pre-PET immunotherapy exposure, as illustrated in Figure 4.

**Figure 4:**
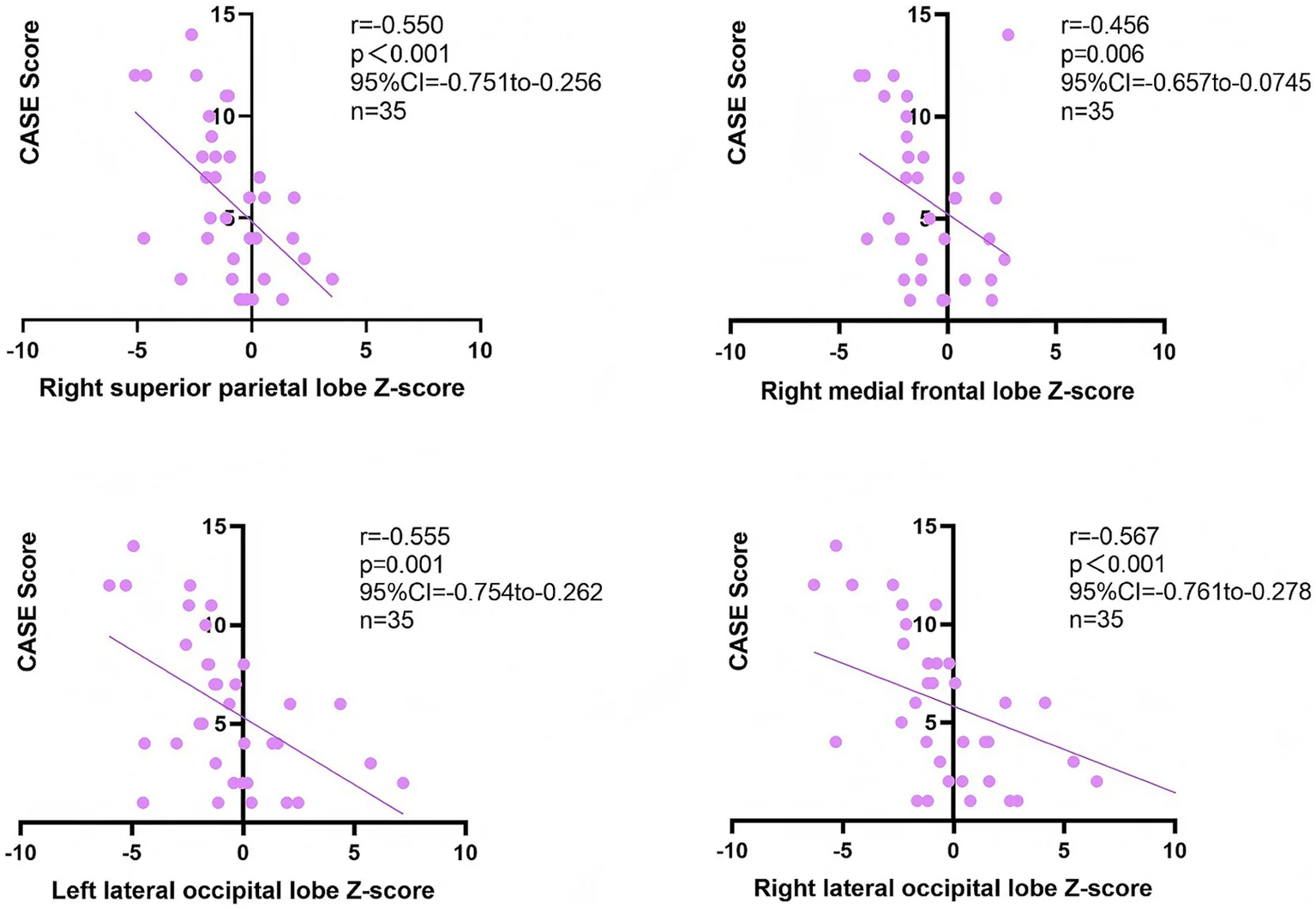
Correlation between Cortex ID Z-score and CASE score. The scatter plot displays simple Spearman correlation coefficients calculated from raw, unadjusted data. The partial correlation results after adjusting for the pre-PET scan immunotherapy status are as follows: right medial frontal lobe, partial *r* = −0.446; 95% CI = −0.662 to −0.072; *p* = 0.010; right superior parietal lobe, partial *r* = −0.582; 95% CI = −0.752 to −0.325; *p* < 0.001; right lateral occipital lobe, partial *r* = −0.463; 95% CI = −0.773 to −0.205; *p* = 0.008; left lateral occipital lobe, partial *r* = −0.519; 95% CI = −0.741 to −0.221; *p* = 0.002.

## 5 Discussion

Encephalitis is an acute neurological condition characterized by inflammatory lesions within the brain parenchyma leading to impaired neurological function. Autoimmune encephalitis (AE), a subset mediated by immune mechanisms, constitutes approximately 20% of all encephalitis cases. In recent years, increased clinical recognition and continuous identification of novel autoantibodies have contributed to a rising diagnostic rate of AE (12).

The subspecialty of neuroimmunological inflammatory diseases is still developing. Due to the often atypical and complex clinical presentations, limited specificity of auxiliary examinations, and suboptimal sensitivity of autoantibody testing, early and accurate diagnosis of AE remains challenging. Timely diagnosis and aggressive treatment are essential to improve prognosis and prevent severe outcomes. This study included 35 patients with clinically diagnosed AE and systematically analyzed their clinical features, laboratory findings, and ^18^F-FDG PET/CT imaging characteristics, with comparative assessment across different levels of disease severity.

Consistent with previous reports, this study found no significant gender predisposition in AE, which can occur across all age groups, typically with acute or subacute onset. Frequent clinical manifestations include psychiatric symptoms, speech impairment, altered consciousness, and sleep disturbances. Previous studies suggest that the neutrophil-to-lymphocyte ratio (NLR) may reflect disease severity and prognosis in AE (13). Our results align with these observations: the moderate-to-severe group exhibited significantly elevated neutrophil percentages and NLR, alongside reduced lymphocyte percentages, lymphocyte counts, and serum calcium levels compared to the mild group. These findings support the potential role of these hematological parameters in evaluating AE severity. Neutrophils, as early responders in autoimmune CNS pathologies, exacerbate inflammation through release of pro-inflammatory cytokines, disrupt the blood–brain barrier, alter the cerebral microenvironment, and contribute to neuronal injury and metabolic dysfunction, ultimately culminating in diverse neurological deficits.

Conventional MRI is widely used in AE diagnosis, typically revealing T2/FLAIR hyperintensities in limbic regions such as the medial temporal lobe, cingulate gyrus, and insula, sometimes extending to subcortical structures (14–16). However, MRI demonstrates limited sensitivity—only 29.4% of patients in our cohort showed abnormalities. A recent systematic review reported ^18^F-FDG PET/CT sensitivity between 80 and 92%, substantially higher than MRI (46–66%) (7). This study demonstrates that ^18^F-FDG PET/CT has a higher abnormality detection rate than brain MRI. ^18^F-FDG PET/CT detected metabolic abnormalities in all 10 patients with MRI-visible lesions, often revealing more extensive involvement. Given that metabolic alterations frequently precede structural changes, ^18^F-FDG PET/CT facilitates the detection of abnormalities more readily. Although the median interval between brain MRI and ^18^F-FDG PET/CT was short (4 days), we acknowledge that temporal gaps between the two imaging modalities may introduce confounding bias, especially when systemic immunotherapy is initiated between scans. Future prospective studies should adopt standardized imaging time-points to minimize such bias.

We further evaluated whether the timing of PET/CT acquisition relative to symptom onset influenced the observed cerebral metabolic patterns. Stratifying patients by the median onset-to-PET interval (26 days) revealed no significant differences in the rates of abnormal PET findings, lobar hypometabolism, or basal-ganglia hypermetabolism between the early- and late-scan groups. Collectively, these findings suggest that, within our observed time window (16–50 days after symptom onset), PET scan timing did not substantially alter the metabolic patterns captured. Several factors may explain this observation. First, all PET examinations were performed during the acute or sub-acute phase of AE, when disease activity remained relatively high. Second, cerebral metabolic abnormalities in AE may remain comparatively stable within this time frame rather than undergoing rapid dynamic shifts. Third, the limited sample size may have reduced the statistical power to detect subtle temporal metabolic changes. Nevertheless, given the cross-sectional design, we cannot evaluate intra-individual longitudinal metabolic evolution over the disease course, and this issue should be addressed in future prospective studies.

The pathophysiology of AE is dynamic. Critically ill patients may undergo a “quiescent phase” of immune activity, during which metabolic abnormalities might not be overt. One severe AE case in our series showed normal FDG-PET, possibly reflecting transient metabolic recovery during scanning or limitations in detecting small or deep-seated foci due to finite spatial resolution. Previous studies consistently report cerebral hypometabolism, particularly in parietal and occipital regions, as a hallmark of AE (5, 17, 18), which aligns with our observations.

In this study, the abnormality detection rates of semi-quantitative analysis using Cortex ID and visual analysis were similar, with discrepancies in some cases. For instance, three cases were assessed as normal by Cortex ID but showed diffuse global hypometabolism on visual analysis. This is because Cortex ID calculates Z-scores using the formula: Z-score = (individual value − control mean)/control standard deviation. Diffuse global hypometabolism may lead to false-negative “individual values” when specific brain regions are used as references, resulting in Z-scores within the normal range and causing the software to classify them as “normal.” This highlights the inherent limitation of voxel-based quantitative analysis in detecting diffuse, symmetric abnormalities. Four cases deemed normal visually showed regional abnormalities on Cortex ID, these 4 clinically mild cases appeared normal on visual assessment; however, Cortex ID identified small regions (<5 voxels) of mild hypermetabolism in limbic regions including the unilateral hippocampus and insula. This reflects the lower sensitivity of visual reading for subtle, focal metabolic changes. Discordance between visual assessment and Cortex ID-based quantitative analysis indicates internal inconsistency in the original analytical pipeline. Visual interpretation depends on reader experience, whereas automated quantification is limited by algorithmic insensitivity to global diffuse reductions. Each approach has distinct strengths and limitations, and neither method alone fully characterizes the heterogeneous metabolic patterns observed in patients with AE. Integrating both approaches is recommended in clinical practice. It should be emphasized that Cortex ID only outputs quantitative cortical metrics and cannot quantify deep subcortical nuclei; visual assessment remains indispensable to identify subcortical metabolic changes such as basal-ganglia hypermetabolism in AE patients.

Previous studies have largely focused on antibody-positive AE as the primary study population. Metabolic patterns on ^18^F-FDG PET/CT vary among antibody subtypes of AE (19). Anti-NMDAR encephalitis typically shows parieto-occipital hypometabolism with frontal/basal ganglia hypermetabolism (18, 20, 21); anti-CASPR2 encephalitis exhibits basal ganglia hypermetabolism (22); LGI1-related AE shows medial temporal or basal ganglia hypermetabolism (23–25); and anti-GAD65 encephalitis is associated with medial temporal hypometabolism (7). Prospective studies with larger cohorts are needed to delineate antibody-specific metabolic profiles comprehensively.

Furthermore, ^18^F-FDG PET/CT parameters show promise in assessing disease severity and prognosis. Pretreatment metabolic lesion burden correlates with modified Rankin Scale scores pre- and post-treatment, with greater cortical involvement predicting poorer recovery (26). Hypermetabolism in the medial temporal lobe may serve as a prognostic biomarker in anti-GABABR encephalitis (27). In our study, Z-scores in frontal, parietal, and occipital regions negatively correlated with CASE scores, suggesting that metabolic dysfunction in these regions may influence AE severity and warrant early intervention. Previous studies have largely focused on qualitative diagnostic performance. In contrast, this study is the first to perform a quantitative correlation analysis between the severity of PET metabolic abnormalities (quantified by Z-scores) and a validated clinical severity scale (CASE score), exploring the novel question of “whether the degree of hypometabolism is related to clinical severity.” Furthermore, this study was conducted in a mixed cohort containing 54.3% antibody-negative patients, which more closely reflects real-world clinical practice compared to previous studies that mostly focused on specific antibody-positive subtypes. It should be emphasized that we did not formally adjust for multiple comparisons in the correlation analyses. Given the limited sample size, these correlation findings should be interpreted with caution and regarded as hypothesis-generating rather than definitive; further validation is warranted in larger prospective studies with adequate control for confounders.

Although ^18^F-FDG is currently the most commonly used PET tracer for evaluating cerebral metabolism in AE, it reflects non-specific alterations in neuronal function and cannot directly visualize core pathological processes such as neuroinflammation or blood–brain barrier disruption. In recent years, novel tracers targeting neuroinflammation have shown considerable potential. For example, the translocator protein (TSPO), which is significantly upregulated on the mitochondrial membrane of activated microglia and macrophages, serves as a key marker of neuroinflammation. Second- and third-generation TSPO tracers (e.g., ^11^C-PK11195, ^18^F-DPA-714, ^18^F-GE180) have been widely used for neuroinflammation imaging in conditions such as multiple sclerosis, Alzheimer’s disease, and stroke (28, 29). A recent study applying ^18^F-DPA-714 PET-MRI in patients with anti-LGI1 and anti-CASPR2 limbic encephalitis reported significantly increased tracer uptake in the hippocampus and amygdala, which correlated with the distribution of activated microglia detected by immunohistochemistry (30). Latest research indicates that the monoamine oxidase B-targeting tracer ^18^F-fluorodeprenyl-D2 (^18^F-F-DED) can assess reactive astrogliosis. In patients with GAD65 antibody-positive AE, its uptake pattern correlates with clinical phenotypes (limbic encephalitis, cerebellar ataxia) and disease severity (31). Although these novel tracers are more mechanistically specific, their clinical availability, degree of standardization, and cost-effectiveness remain far inferior to those of FDG. Therefore, FDG-PET will remain the mainstream tool for imaging assessment of AE for the foreseeable future, while novel tracers hold promise for more refined pathophysiological subtyping or treatment monitoring in the years ahead.

The limitations of this study are as follows: First, this was a retrospective study with potential selection bias. Second, due to the low prevalence of autoimmune encephalitis and the high cost of ^18^F-FDG PET/CT, the sample size was relatively small, which precluded controlling for key confounding factors and resulted in a certain degree of inherent fragility in the statistical findings. Third, the semi-quantitative image analysis method has limitations, as the Cortex ID software only reflects cerebral cortical metabolism and cannot analyze the basal ganglia or other deep brain structures. Fourth, because the sample sizes for each antibody subtype subgroup were too small to conduct adequately powered subtype-stratified analyses, we performed a pooled analysis across all subtypes. This analytical strategy may obscure unique metabolic signatures associated with distinct antibody subgroups and thus limit the ability to interpret findings based on subtype-specific pathophysiological patterns. Fifth, some patients (particularly those in the moderate-to-severe group who may have initiated treatment earlier due to clinical necessity) had already started immunotherapy before undergoing the PET scan. This prior treatment could potentially alter cerebral metabolic patterns. Although our partial correlation analysis adjusting for immunotherapy status confirmed the robustness of the core associations, the binary coding of treatment status cannot fully capture nuanced differences in treatment timing, duration, or specific regimens. Sixth, although the median interval between brain MRI and PET/CT was short (4 days), temporal gaps between the two imaging modalities may introduce confounding bias, especially when systemic immunotherapy is initiated between scans. Seventh, this study only enrolled patients with confirmed AE and did not include a non-AE control group; therefore, it was not possible to calculate the true diagnostic sensitivity, specificity, or predictive values of PET. The comparison between PET and MRI is limited to describing the abnormality detection rate and does not represent a direct comparison of diagnostic performance.

In conclusion, in this retrospective cohort of 35 patients with autoimmune encephalitis, clinical and laboratory parameters, including neutrophil percentage, lymphocyte count, NLR, and serum calcium, were associated with disease severity. ^18^F-FDG PET/CT showed a significantly higher abnormality detection rate than brain MRI. Regional hypometabolism in the frontal, parietal, and occipital lobes was negatively correlated with CASE scores, as quantified by Cortex ID Z-scores. These findings support the use of ^18^F-FDG PET/CT for evaluating disease severity and early diagnosis in AE. However, further validation is needed in larger prospective, multicenter studies.

## Data Availability

The raw data supporting the conclusions of this article will be made available by the authors, without undue reservation.
